# Activation leads to a significant shift in the intracellular redox homeostasis of neutrophil-like cells

**DOI:** 10.1016/j.redox.2019.101344

**Published:** 2019-10-13

**Authors:** Kaibo Xie, Marharyta Varatnitskaya, Abdelouahid Maghnouj, Verian Bader, Konstanze F. Winklhofer, Stephan Hahn, Lars I. Leichert

**Affiliations:** a Ruhr University Bochum, Institute of Biochemistry and Pathobiochemistry, Microbial Biochemistry, Bochum, Germany; b Ruhr University Bochum, Department of Molecular Gastrointestinal Oncology, Bochum, Germany; c Ruhr University Bochum, Institute for Biochemistry and Pathobiochemistry, Molecular Cell Biology, Bochum, Germany

## Abstract

Neutrophils produce a cocktail of oxidative species during the so-called oxidative burst to attack phagocytized bacteria. However, little is known about the neutrophils' redox homeostasis during the oxidative burst and there is currently no consensus about the interplay between oxidative species and cellular signaling, e.g. during the initiation of the production of neutrophil extracellular traps (NETs). Using the genetically encoded redox sensor roGFP2, expressed in the cytoplasm of the neutrophil-like cell line PLB-985, we saw that stimulation by both PMA and *E. coli* resulted in oxidation of the thiol residues in this probe. In contrast to the redox state of phagocytized bacteria, which completely breaks down, the neutrophils' cytoplasmic redox state switched from its intital -318 ± 6 mV to a new, albeit higher oxidized, steady state of -264 ± 5 mV in the presence of bacteria. This highly significant oxidation of the cytosol (p value = 7 × 10^-5^) is dependent on NOX2 activity, but independent of the most effective thiol oxidant produced in neutrophils, MPO-derived HOCl. While the shift in the intracellular redox potential is correlated with effective NETosis, it is, by itself not sufficient: Inhibition of MPO, while not affecting the cytosolic oxidation, significantly decreased NETosis. Furthermore, inhibition of PI3K, which abrogates cytosolic oxidation, did not fully prevent NETosis induced by phagocytosis of bacteria. Thus, we conclude that NET-formation is regulated in a multifactorial way, in part by changes of the cytosolic thiol redox homeostasis in neutrophils, depending on the circumstance under which the generation of NETs was initiated.

## 1 Introduction

Neutrophils are the most abundant circulating granulocytes in the human body. As the first defenders of our immune system, neutrophils attack pathogens by several means. Upon encounter, pathogens such as bacteria are engulfed and internalized into compartments in neutrophils, a process called phagocytosis. As the phagosome matures into the phagolysosome by fusion with different intracellular granules, encapsulated bacteria are attacked by a mixture of toxic molecules including antimicrobial proteins and potent oxidants [1]. The production of reactive oxidants within the phagolysosome is initiated by assembly and activation of the membrane complex NADPH oxidase 2 (NOX2) [2,3]. Activated NOX2 transfers electrons from NADPH to phagosomal oxygen, which generates superoxide anion (O_2_^•-^). Oxidants derived from this radical include hydrogen peroxide (H_2_O_2_) and the hydroxyl radical (^•^OH). H_2_O_2_ reacts further with chloride to form HOCl, a highly reactive oxidant, in a reaction catalyzed by myeloperoxidase (MPO) [4,5]. The activity of NOX2 is known to be essential for killing of microbes. Individuals suffering from chronic granulomatous disease (CGD), a hereditary disease in which NOX2 is inactive, are highly susceptible to microbial infections [6]. Oxidants produced downstream of NOX2 can directly react and thus oxidatively damage cellular components of trapped microbes [[7], [8], [9]].

A growing body of evidence highlights NOX2-related oxidants also as important signaling molecules to regulate cellular functions [[10], [11], [12], [13]]. As such, NOX2 as well as MPO activity was shown to be involved in the activation of the formation of neutrophil extracellular traps (NETs), another crucial antimicrobial mechanism in neutrophils [[14], [15], [16], [17]].

Due to the transient nature of the phagosomal environment, quantitative redox measurements have proven to be difficult [18]. Conventional methods include HPLC quantification of redox pairs after cell disruption and the use of redox-active fluorogenic dyes such as the widely used 2′,7′-dihydrodichlorofluorescein (H_2_DCF) [[19], [20], [21], [22]]. However, those approaches often lack specificity, are prone to photobleaching or can simply not be used for subcellular dynamic measurement in living cells [[23], [24], [25]]. Many of those limitations were overcome by genetically encoded redox sensors. roGFP2, a variant of the enhanced green fluorescent protein (EGFP) has been widely used to study redox dynamics in various cell compartments across different organisms [[26], [27], [28], [29], [30]]. Like in EGFP, the chromophore of roGFP2 is formed by the cyclization of the residues 65–67 (Thr-Tyr-Gly). In close proximity to the chromophore are two engineered cysteine residues (C147 and C204). When they form a disulfide bond, a reversible conformational change in roGFP2 promotes the protonation of Tyr66. roGFP2 emits light at 510 nm and has two excitation maxima at 488 nm and 405 nm respectively [28,31]. Oxidation of C147 and C204 increases the excitation peak at 405 nm at the expense of the excitation peak at 488 nm. The redox states of roGFP2 can thus be measured by a ratiometric determination of its emission intensity at 510 nm at the excitation wavelengths 405 and 488 nm [28,32].

In our study, we developed a neutrophil-like cell line (based on PLB-985) that expresses the genetically-encoded redox sensor roGFP2 in the cytoplasm. This gave us a tool to analyze the redox dynamics in neutrophil-like cells upon activation by external stimuli such as PMA and during physiological events, such as phagocytosis of bacteria. Both PMA and phagocytosis of bacteria led to substantial roGFP2 oxidation, showing that, upon stimulation, the cytoplasmic redox homeostasis of neutrophils shifts to a more oxidizing environment. It also allowed us to study the involvement of oxidation events in the induction of NET-formation through both PMA exposure and bacterial phagocytosis. Our data suggests that the observed cytoplasmic redox-shift by itself is not sufficient to induce NET-formation, but additional components dependent on MPO activity and PKC signaling are required.

## 2 Experimental procedures

### 2.1 PLB-985 cell culture and differentiation

The human myeloid leukemia cell line PLB-985 (DSMZ, German collection of microorganisms and cell culture) was cultured and differentiated as described before [33,34]. In short, cells were cultured in RPMI 1640 medium supplemented with 10% heat-inactivated fetal bovine serum (FBS), 1% GlutaMAX (Life Technologies, Darmstadt, DE) at 37 °C and 5% CO_2,_ passaged twice a week. For granulocytic differentiation of cells, exponentially growing cells at a density of 2 × 10^5^/ml were cultured in RPMI 1640 medium supplemented with 10% FCS, 1% GlutaMAX and 1.25% DMSO for five days. On day four, cells were stimulated with 2000 U/ml interferon-γ (IFN-γ, Immunotools, Friesoythe, DE).

### 2.2 Generation of genetically encoded roGFP2 for expression in PLB-985

For the construction of PLJM1-roGFP2 that was used for the expression of roGFP2 in PLB-985, the gene region encoding roGFP2 was amplified using the primers listed in Supplementary Table 1 from pCC_roGFP2, which served as template (Supplementary Table 1). The PCR products were cloned into PLJM1-EGFP using the restriction enzymes *Bam*Hl and *Nsi*l. *E. coli* stbl3 served as a cloning host and was subsequently used to amplify plasmid DNA. Endotoxin-free plasmids were obtained using the plasmid isolation kit NucleoBond Xtra Midi Plus EF according to the manufacturer's instruction (Macherey-Nagel, Düren, DE). The genomic integrity of roGFP2 was confirmed by restriction analysis and Sanger sequencing (Microsynth Seqlab, Göttingen, DE).

### 2.3 Transduction of PLB-985

The roGFP2 expressing PLB-985 cell line was established using a lentiviral transduction system of the 2nd generation with a single packaging plasmid encoding the *gag*, *pol*, *rev*, and *tat* genes [35]. Viral particle was produced by transfecting the roGFP2 containing plasmid PJLM1-roGFP2 along with the packaging plasmids pCMV-VSV-G and pCMVR8.2 into HEK-293T cells (Supplementary Table 1). 2.5 × 10^6^ cells were seeded on a 10 cm^2^ cell culture dish and cultured in Dulbecco's modified Eagle's medium (DMEM) (Life Technologies, Darmstadt, DE) supplemented with 10% FBS (Life Technologies, Darmstadt, DE) and 1% Penicillin/Streptomycin (PenStrep, Life Technologies, Darmstadt, DE) at 37 °C and 5% CO_2_. After 24 h, for each 10 cm^2^ culture dish, 6 μg of pCMV-VSV-G, 12 μg of pCMVR8.2 and 12 μg of PJLM1-roGFP2 were mixed with H_2_O to a final volume of 438 μl and supplemented with 62 μl of 2 M CaCl_2_. 500 μl of 2xHBS phosphate buffer was added to the mixture dropwise. Afterwards, the solution was incubated for 10 min at RT and added to the HEK-293T cells. After 16 h of incubation at 37 °C and 5% CO_2_, DMEM was exchanged by RPMI supplemented with 10% FCS and 1% GlutaMAX. Those cells were incubated at 37 °C and 5% CO_2_ to allow production of lentiviral particles for 16 h. Viral particles were collected by harvesting the supernatant and pressing it through a 0.45 μm syringe filter (Filtropur s0.45, Sarstedt, Nürnbrecht, DE). To increase the virus titer, viral particles were concentrated to ¼ of the initial volume using an ultrafiltration unit (VIVASPIN 20, 100,000 MWCO PES, Sartorius, Göttingen, DE) at 3000 g and 4 °C. For transduction of PLB-985 cells, 5 ml of concentrated viral particles containing 4 μg/ml Polybrene (Sigma, Darmstadt, DE) was used to resuspend 2 × 10^6^ of PLB-985 cells, which were seeded 24 h prior to transduction. Cells were incubated for 16 h at 37 °C and 5% CO_2_ to allow transduction. Then, the culture media was exchanged with RPMI and the cells were incubated for another 72 h to allow protein expression. roGFP2 expression was analyzed by fluorescence microscopy (IX50, Olympus, DE). Pictures were taken with a SLR camera (Olympus, DE) and the CellP software (Olympus, DE). Those cells were further used for the generation of monoclonal cultures.

### 2.4 Generation of monoclonal culture by FACS

Monoclonal cultures of roGFP2 expressing PLB-985 cells were generated using a fluorescence-activated cell sorter (FACS, BD FACSAria III, BD, Franklin lakes, USA) and the respective software BD FACSDiva (version 8.0.1, BD, Franklin lakes, USA). For this purpose, cells were washed once with PBS (pH 7.4) and resuspended in PBS (pH 7.4) at approximately 10^6^ cells/ml. Then the cells were analyzed to determine the gating parameters for positive clones. In total, 96 GFP-positive clones were sorted into a 96-well plate (Sarstedt, Nürnbrecht, DE) in a single cell mode using the excitation wavelength at 488 nm with a 530/30 emission filter. Single cells generated this way were kept at 37 °C and 5% CO_2_ in RPMI supplemented with 1% GlutaMAX (Life Technologies, Darmstadt, DE) and 30% FBS. The development of single viable cells to single colonies was monitored microscopically and subcultured in RPMI supplemented with 1% GlutaMAX and 10% FBS.

### 2.5 Coomassie staining and Western Blot

Protein samples were separated on precast 4–12% Bis-Tris gels (NuPAGE™, invitrogen, USA) under reducing conditions (200 V, 40 min). Proteins were stained using Coomassie R-250 as described by Wong et al. [36]. In short, gels were boiled in “Fairbanks A″ solution (25% isopropanol, 10% glacial acetic acid, 0.05% Coomassie R-250) followed by “Fairbanks B” (10% isopropanol, 10% glacial acetic acid, 0.0005% Coomasie R-250) and “Fairbanks C” (10% glacial acetic acid, 0.002% Coomassie R-250). For removal of Coomassie, gels were destained in “Fairbanks D” (10% glacial acetic acid). For immunodetection, the iBlot™ 2 Dry Blotting System (Invitrogen, USA) was used to first transfer proteins onto nitrocellulose membranes. Then, those membranes were blocked with 5% milk powder (Sigma, Darmstadt, DE) and incubated with antibodies against GFP (rabbit, 1:4000, Thermo Fisher Scientific, Waltham, USA). Binding of primary antibody was detected with a fluorescent secondary anti-rabbit antibody (goat, 1:10000, IRDye 680RD, LICOR, NE). Blots were visualized using an infrared imaging system (Odyssey Classic, LICOR, NE) with 3 min exposure time.

### 2.6 Real-time analysis of roGFP2 oxidation state in PLB-985 cells

The redox state of roGFP2 in PLB-985 was measured in a 96-well format as described by Degrossoli et al. with minor modifications [37]. In short, 50 μl of roGFP2 expressing PLB-985 cells at a concentration of 10^7^ cells/ml were incubated with respective inhibitors as described (100 nM Wortmannin; 10 μM Diphenyleneiodonium chloride (DPI); 500 μM 4-aminobenzoic acid hydrazide (ABAH); 1 μM Gö 6983 (Gö)) (Sigma, Darmstadt, DE) or in case of control, with PBS for 1 h at 37 °C in a 96-well plate (Nunc black, clear-bottom, Rochester, NY). Afterwards, 50 μl of *E. coli* at an OD_600_ of 1.0 as well as the respective stimulants were added. The fluorescence intensity was recorded every minute for 2 h at the excitation wavelength 405 nm and 488 nm, unless described otherwise. The emission wavelength was set to 510 nm. The Calculation of the 405/488 nm ratio was done using Microsoft Excel 2016 (Microsoft, USA). Visualization of respective graphs were done using GraphPad Prism (version 5.00, USA). All plate reader assays were performed in at least three independent experiments. The end point ratio, as depicted in the bar graphs, was calculated based on the final point of a linear regression over the last 10 min of the measurement.

### 2.7 Determination of the redox potential of roGFP2 in PLB-985

The redox potential of roGFP2 expressed in the cytoplasm of PLB-985 neutrophil-like cells was calculated as described in previous studies [29,38,39]. Fluorescence intensities were measured in PBS, pH 7.4. Oxidized roGFP2 was generated using 2 mM Aldrithiol-2 (AT-2) and reduced via 50 mM Dithiothreitol (DTT) respectively. The degree of roGFP2 oxidation (OxD_roGFP2_) was calculated using the following formula:

$$OxD_{roGFP2}=\frac{R-R_{red}}{\frac{I_{max}}{I_{min}}*\left(R_{ox}-R\right)+\left(R-R_{red}\right)}$$

In this equation, R_ox_ is the 405/488 ratio of oxidized roGFP2 and R_red_ of reduced roGFP2 respectively. I_max_ and I_min_ are the absolute fluorescence intensities of roGFP2 at 488 nm under reduced and oxidized conditions. R is the 405/488 nm ratio of roGFP2 in PBS (7.4) (R_medium_) or upon stimulation of PLB-985 cells by PMA (R_PMA_) and *E. coli* (R_*E.coli*_). For the calculation of R_medium_, the measured 405/488 values over a time course of 120 min were fitted using linear regressions. R_medium_ was calculated from the respective fitted equation at the first measured time point (T = 1). Accordingly, the 405/488 values of the last 40 min of measurement upon stimulation by *E. coli* and PMA were used to generate equations of linear regressions, from which the last measurement point (T = 40) was used to calculate R_*E.coli*_ and R_PMA_. OxD_roGFP2_ is used to calculate the intracellular redox potential of roGFP2 E_roGFP2_ as follows:

$$E_{\mathrm{roGFP}2}=E_{roGFP2}^{o^{'}}-\frac{R*T}{n*F}*\text{ln}\left(\frac{1-{\text{OxD}}_{\text{roGFP}2}}{{\text{OxD}}_{\text{roGFP}2}}\right)$$

E^o'^_roGFP2_ is -280 mV [28], *R* is the gas constant (8.314 J K^-1^mol^-1^), *T* is the temperature (310.15 K), *n* is the number of transferred electrons (2) and *F* is the Faraday's constant (96,485 C mol^-1^).

### 2.8 Phagocytosis of bacteria by PLB-985 cells

Cultures of *E. coli* harbouring pASK-IBA3 containing mCherry or alternatively pCC LV (Supplementary Table 1) was grown to an OD_600_ of 0.5 at 37 °C with 100 μg/ml ampicillin. For mCherry expression overnight at 20 °C, 100 μM Isopropyl-β-D-thiogalactopyranosid (IPTG) were added. Bacteria were washed twice in PBS (pH 7.4) and opsonized with 5 mg/ml human immunoglobulin G (hIgG, Sigma, Darmstadt, DE) for 30 min at 37 °C. Then, bacteria were washed twice with PBS and resuspended in PBS supplemented with 0.5% FBS to an OD_600_ of 1 (~10^9^ cells/ml), unless described differently. Differentiated PLB-985 cells, which stably expressed roGFP2 in the cytoplasm, were washed once with PBS, resuspended in PBS supplemented with 0.5% FBS to a concentration of 10^7^ cells/ml and mixed with opsonized *E. coli* in the same volume (multiplicity of infection, MOI = 100) to start phagocytosis.

### 2.9 Fluorescence live-cell imaging with subsequent ratiometric image analysis

The fluorescence live-cell imaging of roGFP2 oxidation in PLB-985 cells was performed as described previously [37]. Differentiated PLB-985 cells with roGFP2 stably expressed in the cytoplasm were washed once with PBS and diluted in PBS with 0.5% FBS to a final concentration of 10^7^ cells/mL. 1 mL of the PLB-985 cell suspension was mixed with opsonized *E. coli* cells with a ratio of one PLB-985 cell to 100 *E. coli* in an imaging dish (μ-Dish 35 mm, high, Ibidi, DE). Fluorescence images were acquired using the LSM 880 ELYRA PS.1 microscope (Carl Zeiss Microscopy GmbH, Jena, DE). Images were acquired in different channels according to the fluorophore, Ex_405nm_/Em_513nm_ and Ex_488nm_/Em_513nm_ to detect roGFP2; Ex_561nm_/Em_610nm_ to detect mCherry; Ex_633nm_/Em_645nm_ to detect Alexa Fluor 647 and Ex_405nm_/Em_442nm_ to detect Hoechst 33342. Individual single channel images were exported using ZEN 2.1 (Zeiss, DE). Individual cells were detected using the function “MeanROI view” in the ZEN software and calculation of the 405/488 nm ratio was done based on the “mean intensity measurement” over the surface of the respective cell. For the assembly of ratiometric time series, images were smoothed and subtracted from background. The normalized 405/488 nm ratio image series were exported directly as pictures or assembled to a movie using a software kindly provided by Mark Fricker [40].

### 2.10 Visualization and quantification of NET formation

Microscopic visualization of NET formation was performed as described by Brinkmann et al. with minor modifications [41]. Briefly, 250 μl of differentiated PLB-985 in PBS (pH 7.4) with 0.5% FCS were seeded on 12 mm non-coated coverslips (High precision, thickness 170 μm, Paul Marienfeld, Lauda-Königshofen, DE) that were laid into a 24 well cell culture plate (Sarstedt, Nürnbrecht, DE). These cells were incubated with or without inhibitors for 1 h at 37 °C prior to stimulation with 250 μl of 250 nM PMA or *E. coli* (MOI = 100). After 4 h of incubation at 37 °C, cells were fixed with 4% paraformaldehyde for 15 min at RT, permeabilized with 0.25% Triton X-100 (Sigma, Darmstadt, DE) for 10 min, washed 2x with PBS and blocked with 5% bovine serum albumin (Sigma, Darmstadt, DE) overnight at 4 °C. Immunostaining was performed with mouse anti-DNA/Histone1 antibody (1:1000, MAB 3864, Merck, Darmstadt, DE) for 1 h at RT followed by an Alexa Fluor 647–conjugated goat anti-mouse antibody (1:1000, A-21235, Thermo Fisher Scientific, Waltham, USA) for 1 h at RT. Coverslips were stained with Hoechst 33342 (0,6 μg/ml, Thermo Fisher Scientific, Waltham, USA) for 10 min under low-light conditions. Then, coverslips were washed 2x with PBS (pH 7.4) and mounted in ProLong Diamond antifade mountant (Thermo Fisher Scientific, Waltham, USA). Samples were visualized in a Zeiss LSM880 ELYRA PS.1 microscope (Carl Zeiss Microscopy GmbH, Jena, DE). Fluorescence images were exported using ZEN 2.1 (Carl Zeiss Microscopy GmbH, Jena, DE). Quantification of NETosis rate was performed as described by Brinkmann et al. [42]. Briefly, using ImageJ 1.51e (National Institutes of Health, USA), fluorescence images were transferred to an 8-bit format, thresholded and converted to a binary mask. Cell numbers were counted automatically using the function “analyze particles”. Subsequently, NETosis rate (%) was calculated as the ratio of number of neutrophils heavily stained by anti-DNA/Histone1 antibody, and thus underwent NETosis, divided by the total cell number, as visualized by the merged channel of GFP and anti-DNA/Histone1 (see Figure supplement 1). These experiments were performed in biological triplicates.

## 3 Results

### 3.1 Expression of roGFP2 in PLB-985 cells

Professional phagocytic immune cells such as neutrophils activate NOX2 when they encounter invading pathogens. Activation of NOX2 is accompanied by a broad range of different cellular responses, chief amongst them the production of different reactive oxidants. In previous studies, we observed that this oxidant production, particularly the production of HOCl, leads to a total breakdown of the thiol redox state of bacteria phagocytized by a neutrophil-like cell line [37,43]. We were interested if a similar disturbance in the thiol redox state can also be observed in the host cell, after all, the only barrier between the oxidants produced downstream of NOX2 is the membrane of the phagolysosome.

**Fig. 1:**
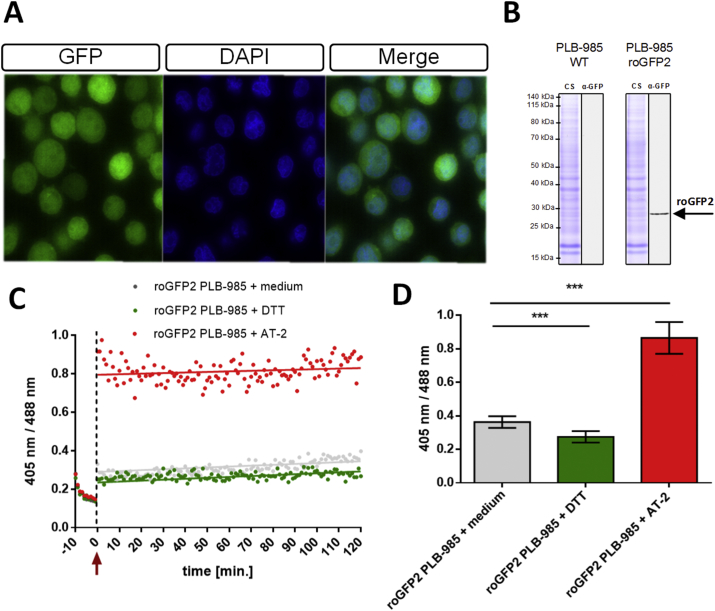
**Expression of roGFP2 in the cytoplasm of the myeloid cell line PLB-985. Redox-sensing activity of roGFP2 was assessed fluorometrically. (A)** Expression of genetically encoded roGFP2 in PLB-985 cells using a lentiviral transduction system as visualized by fluorescence microscopy. Monoclones of transfected cells show homogenous expression of roGFP2. **(B)** PLB-985 cells that expressed roGFP2 (PLB-985 roGFP2) were lysed and the extract containing 10 μg protein was separated by SDS-PAGE. Proteins were stained by Coomassie (CS) and detected by Western Blot with an anti-GFP antibody (α-GFP). PLB-985 wild type cell served as the control (PLB-985 WT). **(C)** The response of roGFP2 to the thiol oxidant aldrithiol-2 (AT-2) and the reductant dithiothreitol (DTT) was measured using a fluorescence plate reader. The redox state of roGFP2 was monitored based on the ratio of 405/488 for 120 min. The arrow points to the time point of the addition of AT-2, DTT or medium. **(D)** Bar graph depicting the ratio at 405 nm/488 nm at the end of the experiment. Based on these values, a steady-state redox potential of roGFP2 in the cytoplasm of PLB-985 of -318 ± 6 mV was established. C: representative results, D: mean values ± SD of at least 3 independent experiments. Significance was calculated using Student's t-test ***: p < 0.001.

For our experiments, we used the myeloid PLB-985 cell line that can undergo granulocytic differentiation, showing highly similar properties to PMNs [34,44]. Using a lentiviral transduction system, we could stably express the genetically-encoded redox-sensor roGFP2 in the cytoplasm of PLB-985. Since transduction results in variable numbers of lentivirus integration [45], single clones expressing roGFP2 were sorted into 96-well plates using fluorescence-assisted cell sorting (FACS). One monoclonal cell population generated this way was used for our experiments. The expression of roGFP2 was confirmed by fluorescence microscopy and Western Blot analysis (Fig. 1A and B). Next, we determined the ratiometric response range of roGFP2 towards the oxidant AT-2, which should fully oxidize all roGFP2 present in the cell, and the reductant DTT, which should fully reduce roGFP2. Using fluorescence spectroscopy, emission intensities at 510 nm with excitation wavelengths at 405, and 488 nm respectively, were quantified. Upon addition of 1 mM AT-2 to differentiated roGFP2-expressing PLB-985 cells, roGFP2 showed a 405/488 nm ratio of approximately 0.8. In contrast, cells treated with 50 mM DTT showed a 405/488 nm ratio of approximately 0.25. This suggests that roGFP2 expressed in the cytoplasm of neutrophil-like PLB-985 cells is indeed redox-sensitive. Untreated control cells showed a 405–488 nm ratio comparable to DTT-treated roGFP2, demonstrating an overall reduced state of roGFP2 in resting PLB-985 cells, in agreement with our expectations (Fig. 1C) [32,46].

Based on the ratio of 405–488 nm under control conditions and the known standard redox potential E^0^_roGFP2_ = −280 mV of roGFP2, we were able to calculate an E_roGFP2_ of -318 ± 6 mV. Assuming that roGFP2 is in equilibrium with cytoplasmic glutathione [23], this should reflect the steady state redox potential of a resting neutrophil-like cell.

### 3.2 NOX2 activation leads to roGFP2 oxidation

**Fig. 2:**
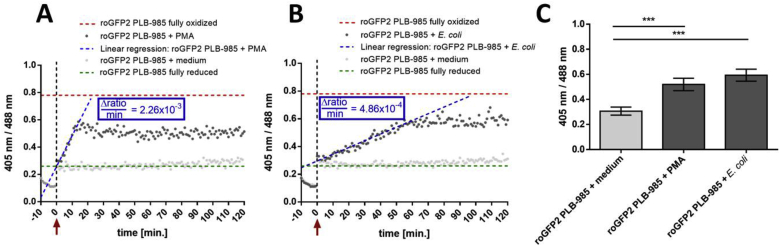
**Fluorometric real-time measurement of roGFP2 expressing PLB-985 neutrophil-like cells upon PMA-stimulation and during *E. coli* phagocytosis.** PLB-985 neutrophil-like cells expressing roGFP2 were mixed with PBS, 250 nM PMA **(A)** or *E. coli* (Multiplicity of infection: one PLB-985 cell to one hundred *E. coli)***(B)** at the time indicated by the red arrow and the non-axial dotted vertical line. The probe's full oxidation (red dotted line) and reduction (green dotted line) in PLB-985 cells was established by incubation with 1 mM AT-2 and 50 mM DTT respectively. The fluorescence intensity at both the excitation wavelengths 488 nm and 405 nm was monitored over 120 min in a 96-well plate reader. Once PMA was added, the 405/488 nm ratio increased promptly from 0.3 to 0.6 and reached its maximum after 20 min, for which the change in 405/488 nm ratio value per min was calculated to be 2.26 × 10^-2^ (blue dotted line). When mixed with *E. coli*, the 405/488 nm ratio increased from 0.3 to 0.6 in a time-dependent manner and reached its maximum after 60 min, for which the change in 405/488 nm ratio value per min was calculated to be 4.86 × 10^-3^ (blue dotted line). **(C)** Bar graph depicting the ratio at 405 nm/488 nm at the end of the experiment under the respective conditions. A and B: representative results, C: mean values ± SD of at least 3 independent experiments. Significance was calculated using Student's t-test ***: p < 0.001. (For interpretation of the references to color in this figure legend, the reader is referred to the Web version of this article.)

To test if intrinsic generation of oxidants leads to a change in the cytosolic redox state of neutrophils, we used Phorbol 12-myristate 13-acetate (PMA). PMA is a synthetic activator of protein kinase C (PKC). Activated PKC then leads to phosphorylation of NOX2 and thus its activation [47]. To measure roGFP2 response upon PMA stimulation, we stimulated differentiated PLB-985 cells with 250 nM PMA and obtained the 405/488 nm ratio using a fluorescence plate reader. An immediate increase of 405/488 ratio indicated a substantial oxidation of roGFP2, reaching a maximum at 20 min. The changes in 405/488 nm ratio per min was calculated for this time frame to be 2.26 × 10^-2^. This was followed by a minor decrease in probe's oxidation between 20 min and 30 min and finally reached a plateau after 30 min of incubation, which stayed at the same level until the end of the measurement (Fig. 2A). This suggests that the cytosol reached a new steady state redox equilibrium. The redox potential of roGFP2 at that steady state was determined to be -267 ± 7 mV, substantially higher than resting cells (p-value _PMA vs Medium_ = 1.6 × 10^-4^).

### 3.3 Phagocytosis of bacteria leads to oxidation of the cytosolic probe

roGFP2, when expressed in *E. coli*, is oxidized within seconds upon phagocytosis of the bacteria by neutrophil-like cells [37]. Here, we monitored the redox state of roGFP2 in PLB-985 neutrophil-like cells during phagocytosis of bacteria. For this, we co-incubated neutrophil-like PLB-985 cells with *E. coli*. The 405/488 nm excitation was then used to determine oxidation state of the roGFP2 expressed in PLB-985. Upon co-incubation with *E. coli*, the 405/488 nm ratio of roGFP2 in the cytosol of neutrophil-like cells increased gradually, reaching a plateau at 70 min that remained at the same level until the end of the measurement. As compared to cells stimulated with PMA, the increase in 405/488 ratio per min was measurably slower in neutrophils co-incubated with *E. coli* and showed a slope of 4.86 × 10^-3^ during the first 70 min. The redox potential of roGFP2 in this oxidized state was calculated to be -264 ± 5 mV (p-value_*E.coli* vs. Medium_ = 7 × 10^-5^), comparable to the oxidation state of roGFP2 in neutrophil-like cells exposed to PMA. When PLB-985 cells were incubated with medium, the 405/488 nm ratio did not significantly change (Fig. 2B).

### 3.4 roGFP2 oxidation in individual neutrophil-like cells occurs within minutes upon phagocytosis of bacteria

**Fig. 3:**
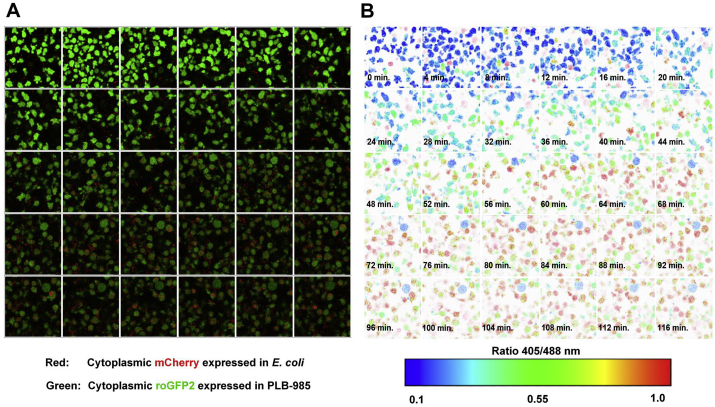
**roGFP2 is gradually oxidized within minutes in phagocytizing PLB-985 neutrophil-like cells. (A)** Fluorescence microscopy shows the uptake of mCherry-expressing *E. coli* cells by roGFP2-expressing PLB-985 neutrophil-like cells over a time course of 116 min. **(B)** The 405/488 nm ratio of roGFP2 in phagocytizing PLB-985 cells was determined using ratiometric quantitative fluorescence microscopy. As illustrated by the false color scale, the oxidation state of roGFP2 from individual cells phagocytizing *E. coli* changed within minutes. Upon stimulation with *E. coli*, the roGFP2 in the majority of the cells within the field of view reached an overall oxidized state after approximately 70 min. Afterwards, the probe's oxidation remained at the same level until the end of the measurement. (For interpretation of the references to color in this figure legend, the reader is referred to the Web version of this article.)

**Fig. 4:**
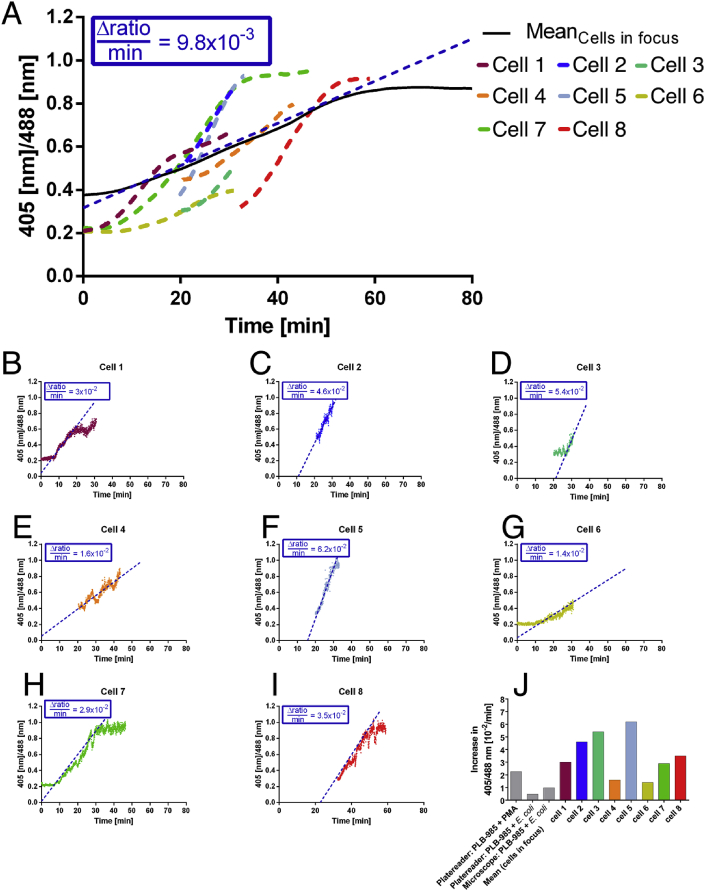
**Quantitative fluorescence microscopy for the ratiometric assessment of roGFP2 oxidation in PLB-985 neutrophil-like cells during *E. coli* phagocytosis. (A)**The 405/488 nm ratio of roGFP2 in phagocytizing PLB-985 cells was determined using quantitative fluorescence microscopy. Excitation intensities at 405 nm and 488 nm were acquired from all PLB-985 cells in focus (black line) or from individual, single cells (dashed colored lines, colors correspond to colors in panels B–I and J). Overall, roGFP2 was gradually oxidized reaching a plateau 70 min after stimulation (cf. Fig. 3 and Video 1). **(B–I)** However, the probe's oxidation in individual cells is not synchronized and the time course of oxidation ranges from 20 to 30 min, resulting in often faster kinetics for individual cells when compared to the average of all cells. (cf. Videos 2-9). Plots in B–I correspond to the identically colored dashed lines in A. **(J)** Velocity of oxidation of all cells in focus compared to selected individual cells. Colors correspond to colors in panels A and B–I. (For interpretation of the references to color in this figure legend, the reader is referred to the Web version of this article.)

In order to verify the changes in 405/488 nm ratio during co-incubation with *E. coli* and in order to gain further insight into the dynamic of the probe's oxidation, we monitored the change of roGFP2 oxidation during phagocytosis using quantitative fluorescence microscopy. Our data showed that the probe was indeed in an overall reduced state in cells that have not taken up *E. coli*. As PLB-985 cells started to phagocytize *E. coli* cells, the redox state of roGFP2 changed gradually into an overall oxidized state (Fig. 3, Video 1). Single cell analysis showed a non-synchronized start of probe oxidation. Once individual neutrophil-like PLB-985 cells phagocytized bacteria, the probe changed its oxidation with up to 6.3 x faster oxidation kinetics (Fig. 4, Videos 2-9). Thus, the gradual increase in probe oxidation measured in the fluorescence plate reader reflects the accumulation of roGFP2 oxidation from single PLB-985 cells that have phagocytized bacteria over time.

Supplementary video related to this article can be found at https://doi.org/10.1016/j.redox.2019.101344

The following are the supplementary data related to this article:

Video 1 3 Video 1

Video.2 Video.2

Video.3 Video.3

Video.4 Video.4

Video.5 Video.5

Video.6 Video.6

Video.7 Video.7

Video.8 Video.8

Video.9 Video.9

### 3.5 roGFP2 oxidation during neutrophil activation is dependent on NOX2, but not on myeloperoxidase activity

**Fig. 5:**
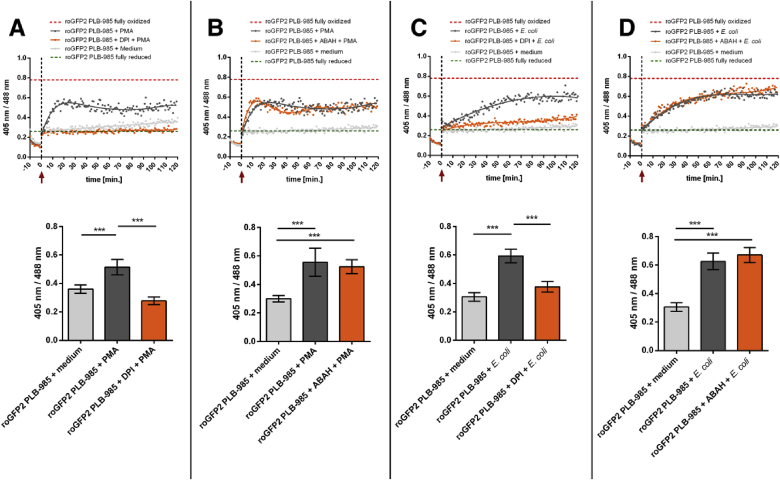
**roGFP2 oxidation is dependent on NOX2-but not MPO-activity.** PLB-985 neutrophil-like cells expressing roGFP2 were pre-treated with the NOX-inhibitor diphenyleneiodonium (DPI) and the MPO-inhibitor 4-aminobenzoic acid hydrazide (ABAH) for 1 h at 37 °C. Then the cells were stimulated as indicated by the red arrow and the non-axial dotted vertical line, with 250 nM PMA (**A, B**) or *E. coli* to a multiplicity of infection of 100 *E. coli* to one PLB-985 cell (**C, D**). The probe's full oxidation (red dotted line) and reduction level (green dotted line) in PLB-985 cells was determined by incubation with 1 mM AT-2 and 50 mM DTT respectively. The fluorescence intensity at both the excitation wavelengths 488 nm and 405 nm was monitored over 120 min in a 96-well plate reader. Pre-treatment with the NOX2-inhibitor DPI abrogated the probe's oxidation. However, Inhibition of MPO by ABAH did not have a significant effect on PMA-induced or *E. coli*-induced oxidation of roGFP2. Scatter plots depict representative results, bar graphs the mean values ± SD of the ratio reached at the end of at least 3 independent experiments under the respective treatments. Significance was calculated using Student's t-test ***: p < 0.001. (For interpretation of the references to color in this figure legend, the reader is referred to the Web version of this article.)

When incubated with *E. coli*, the oxidation of roGFP2 in PLB-985 cells was not as fast as the probe's response in cells that were stimulated with PMA. This is probably due to the asynchronous phagocytosis of individual bacteria as compared to the instantaneous exposure to the molecular stimulant PMA. It could also be caused by a different underlying mechanism of roGFP2 oxidation. Diphenyleneiodonium (DPI), a widely used inhibitor of NOX2 [48], strongly abrogated roGFP2 oxidation during PMA activation. (Fig. 5A). This suggests that PMA leads to roGFP2 oxidation indeed in a NOX2-dependent way. Then, we examined the role of NOX2 in *E. coli*-induced probe oxidation. When pre-treated with the NOX2-inhibitor DPI, the oxidation of roGFP2 in PLB-985 cells during co-incubation with *E. coli* was strongly diminished as well (Fig. 5C). This indicates that roGFP2 oxidation in PLB-985 cells during phagocytosis relies on NOX2 activity, too.

The generation of non-mitochondrial superoxide anion in neutrophils is mainly initiated by NOX2. Superoxide anion is further processed into other products such as the potent oxidant HOCl. Recently, we showed that HOCl generated by myeloperoxidase is the main factor in the oxidation of roGFP2 expressed in bacteria during phagocytosis [37]. As HOCl is known to be a highly effective thiol-oxidant [49], and has been shown to react promptly with roGFP2 in vitro [50], we assessed the role of myeloperoxidase on roGFP2 oxidation in PLB-985. Surprisingly, pre-incubation of neutrophils with the myeloperoxidase inhibitor 4-aminobenzoic acid hydrazide (ABAH) had almost no effect on probe oxidation by PMA stimulation (Fig. 5B) or *E. coli* phagocytosis (Fig. 5D). This suggests that in contrast to phagocytized bacteria, HOCl is not the reactive species that leads to roGFP2 oxidation in the cytoplasm of PLB-985 cells.

### 3.6 Different pathways lead to roGFP2 oxidation in neutrophil-like cells treated with PMA and *E. coli*

**Fig. 6:**
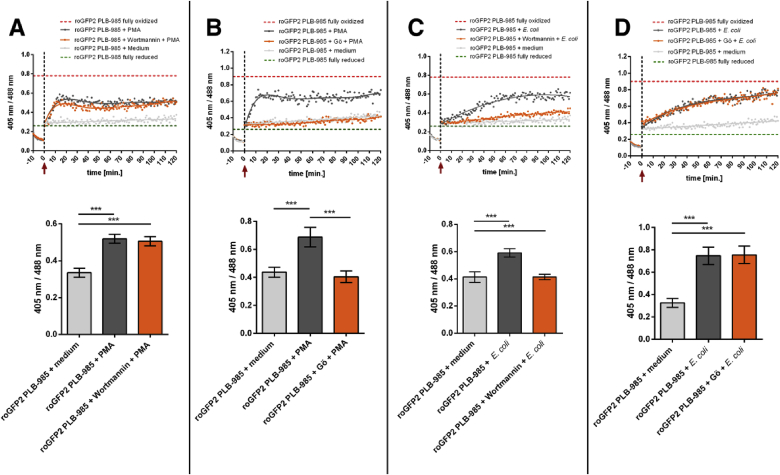
**PMA induces roGFP2 oxidation that is mechanistically distinct from *E. coli***. PLB-985 neutrophil-like cells expressing roGFP2 were pre-treated with the PI3K-inhibitor Wortmannin and the PKC-inhibitor Gö 6983 (Gö) for 1 h at 37 °C. Then the cells were stimulated as indicated by the red arrow and the non-axial dotted vertical line, with 250 nM PMA (**A, B**) or *E. coli* to a multiplicity of infection of 100 *E. coli* to one PLB-985 cell (**C, D**). The probe's full oxidation (red dotted line) and reduction (green dotted line) in PLB-985 cells was determined by incubation with 1 mM AT-2 and 50 mM DTT, respectively. The fluorescence intensity at both the excitation wavelengths 488 nm and 405 nm was monitored over 120 min in a 96-well plate reader. PMA-induced oxidation of roGFP2 is unaffected by the PKC-inhibitor Gö 6983, however, significantly decreased when PI3K-inhibitor Wortmannin was used. *E. coli*-induced probe's oxidation is dependent on activity of PI3Ks but less on PKCs. Scatter plots depict representative results, bar graphs the mean values ± SD of the ratio reached at the end of at least 3 independent experiments under the respective treatments. Significance was calculated using Student's t-test ***: p < 0.001. (For interpretation of the references to color in this figure legend, the reader is referred to the Web version of this article.)

Intruding bacteria are typically marked by opsonins such as IgG-antibodies. Those opsonized bacteria are then recognized by neutrophils via Fc*γ*-receptors (Fc*γ*-Rs). Subsequent phosphorylation of Fc*γ*-Rs leads to the activation of several down-stream signaling molecules. Amongst the enzymes recruited are phosphoinositide 3-kinases (PI3K) and protein kinase C (PKC). Both were shown to be involved during the activation of NOX2 [47,[51], [52], [53], [54], [55], [56], [57]]. To evaluate whether PI3K is required to induce oxidation of roGFP2, roGFP2-expressing PLB-985 cells were incubated with 100 nM of the PI3K inhibitor Wortmannin for 1 h before stimulation [58,59]. Inhibition of PI3K by Wortmannin resulted in a visible attenuation of roGFP2 response upon stimulation with *E. coli* (Fig. 6C). However, the probe's oxidation was not affected when PLB-985 cells were activated by PMA (Fig. 6A). Conversely, pre-inhibition of PKC by the inhibitor Gö 6983 prevented PMA-induced roGFP2 oxidation, as expected, but did not affect probe oxidation during *E. coli* phagocytosis (Fig. 6B, D) [60]. These observations are in line with the fact that PMA leads to NOX2 activation via a PKC-dependent pathway, whereas NOX2 activation caused by *E. coli* typically involves the activation of PI3K [61].

### 3.7 PLB-985 neutrophil-like cells, when activated by PMA or *E. coli*, generate neutrophil-extracellular traps

**Fig. 7:**
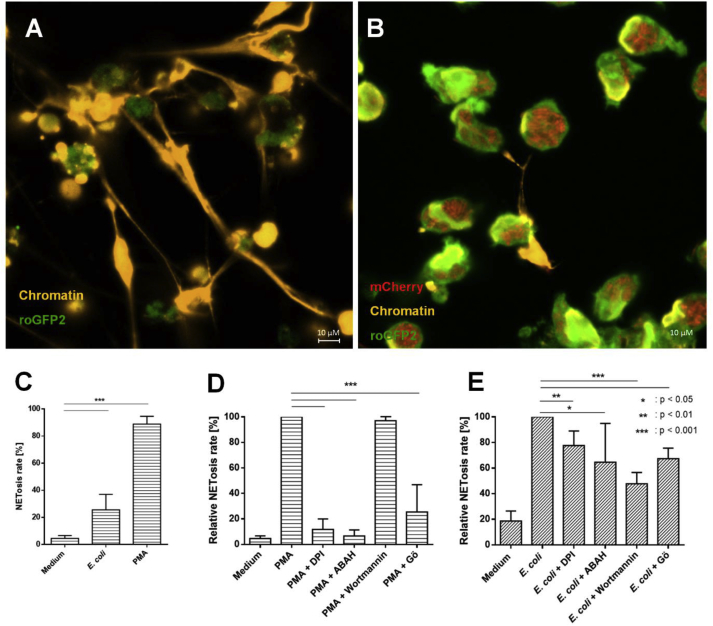
**Formation of NET-formation upon PMA and *E. coli* stimulation and the effect of diphenyleneiodonium chloride (DPI, NOX-inhibitor), 4-aminobenzoic acid hydrazide (ABAH, MPO-inhibitor), Wortmannin (PI3K-inhibitor) and Gö 6983 (Gö, PKC-inhibitor) on the generation of NETs.** roGFP2-expressing PLB-985 neutrophil-like cells were incubated with 250 nM PMA **(A)** or mCherry-expressing *E. coli* cells in a ratio of one hundred PLB-985 cells to one *E. coli***(B)** at 37 °C for 4 h. Extracellular NET-formation is visualized by an antibody directed against chromatin (orange). The formation of NET is expressed by the NETosis-rate, which is calculated based on the ratio of cells heavily stained by the chromatin antibody and the total cell number as described in section 2.1**(C)**. The effect of different inhibitors on formation of NET was assessed by pre-treating PLB-985 cells with the respective inhibitors for 1 h before stimulation. Afterwards, those cells were stimulated with 250 nM PMA **(D)** or *E. coli***(E)** in a ratio of one hundred *E. coli* to one PLB-985 cell for 4 h. NET-producing cells were visualized using an antibody directed against chromatin. The calculation of the NETosis rate was done as described in the materials and methods section. The relative NETosis rate represents the normalized values relative to PMA- and *E. coli*-induced NET formation respectively. NET-formation induced by PMA is abrogated upon inhibition of NOX2, MPO and PKC. The formation of NETs generated upon *E. coli* phagocytosis is significantly inhibited by DPI, ABAH, Wortmannin and Gö 6983. The quantitative results represent mean values ± SD of at least 3 independent experiments. Significance was calculated using Student's t-test (*: p < 0.05, **: p < 0.01, ***: p < 0.001). For raw image data associated with C – E please see linked dataset: figure supplement 1. (For interpretation of the references to color in this figure legend, the reader is referred to the Web version of this article.)

Our data with roGFP2 demonstrated that *E. coli* phagocytosis lead to NOX2 activation in PLB-985 neutrophil-like cells, which resulted in the probe's oxidation. Generation of oxidants is used by neutrophils to attack intruding bacteria. However, oxidants downstream of NOX2 are also thought to serve as signaling molecules. As such, the formation of neutrophil-extracellular traps (NETs) to facilitate phagocytosis and killing of bacteria was shown to be dependent on NOX2-activity [62,63]. To test if the activation of NOX2 indeed leads to NET-formation in PLB-985 cells, we seeded differentiated PLB-985 cells on coverslips and stimulated the cells with PMA or *E. coli* for 4 h. Production of NETs was then visualized by immunofluorescence microscopy using an anti-chromatin antibody. Both PMA and *E. coli* phagocytosis resulted in release of fibers containing decondensed chromatin, which corresponds to NETs (Fig. 7A and B). To quantify NET-formation, we calculated the NETosis-rate based on the percentage of cells heavily stained by an anti-chromatin antibody relative to the total cell number as described by Brinkmann et al. [42]. Approximately 25% of the neutrophil-like cells that were co-incubated with *E. coli* underwent NETosis. In contrast, stimulation with PMA was more effective and resulted in a NETosis-rate of nearly 90% (Fig. 7C).

### 3.8 NET-formation is dependent both on the activity of NOX2 and myeloperoxidase (MPO)

As shown with roGFP2, the inhibition of NOX2 by DPI completely abolished the probe's oxidation after stimulation by both PMA and *E. coli*. However, the probe's oxidation was not affected by the MPO-inhibitor ABAH. To test the effect of NOX2-related oxidants on the ability of PLB-985 cells to form NETs, we pre-treated the cells with both DPI and ABAH for 1 h and determined the NETosis rate after 4 h incubation with PMA and *E. coli*. Both NOX2 and MPO have been reported to be essential for NET formation and we wanted to test if the same was the case in our model setting. Upon activation by PMA, both DPI and ABAH prevented cells from forming NETs (Fig. 7D). When activated with *E. coli*, inhibition of NOX2 and MPO reduced the NETosis rate by 20% and 30% respectively (Fig. 7E). This suggests that the presence of HOCl, produced downstream of NOX2, is an important factor in the mediation of NET-formation, although it has no direct influence on the redox state of the cytosol, as measured by roGFP2.

### 3.9 Signaling pathways involved in NET formation

Having determined that the overall change in the cytosolic redox potential is not the determining factor in NET formation, we took a closer look at the signaling pathways upwards of NOX2. Using the specific inhibitors Gö 6983 and Wortmannin, we again blocked PKC and PI3K activity, respectively. NET formation induced by PMA was unaffected by Wortmannin but was reduced by approximately 70%, when PKC was blocked (Fig. 7D). This suggests that PMA leads to NET formation that is dependent on the activity PKC, comparable to oxidation of roGFP2. This would be still in line with a redox signal directly downstream of NOX2. We then analyzed the ability of neutrophil-like cells to form NETs after co-incubation with bacteria when pre-treated with Gö 6983 or Wortmannin. Inhibition of PI3K reduced NET-formation by 50%. Interestingly, preincubation with Gö 6983 also decreased NET-formation by 30% indicating that redox-independent PKC signaling is involved in NET formation as well (Fig. 7E).

## 4 Discussion

In neutrophils, the enzyme NOX2 plays a key role in the clearance of invading microbes. Oxidants produced downstream of NOX2 are used to attack and kill bacteria either directly or, indirectly, as signaling molecules, activating further immune functions. However, due to the short life span of neutrophils and the transient and complex nature of NOX2-derived oxidants, the acquisition of spatial, temporal and quantitative information about redox processes underlying immune responses have proven to be difficult [23,64,65]. Here, we generated a stable PLB-985 neutrophil-like cell line expressing roGFP2 and used this biosensor to study real-time redox dynamics in the host cell upon stimulation by PMA and during phagocytosis of bacteria.

When expressed in neutrophil-like cells, roGFP2 showed a redox potential of -318 mV. Using the response towards AT-2 and DTT as a measure for the dynamic range of roGFP2 in this cell line, we could show that roGFP2 was initially ~95% reduced in the cytosol of PLB-985 cells, corresponding to the above mentioned E_roGFP2_ of -318 ± 6 mV. This is in agreement with other reports, in which the basal redox potential of roGFP2 expressing HeLa cells was reported to be -325 mV [32]. Given that the main cellular redox buffer glutathione is found in the millimolar range in cells, the oxidation state of roGFP2 has been suggested to reflect the redox state of glutathione (i.e. the ratio and concentration of GSH and GSSG). As such, roGFP2 has been extensively used to measure E_GSH_ in plants, yeast and mammalian cells, which was determined to be between -300 and -320 mV [23,38,46,66,67]. Overall, we can conclude that neutrophil-like cells, when not activated, do have a cytosolic redox potential similar to other cell types.

This, however, changes, once the neutrophil-like cell line is activated. When we measured the response of roGFP2 upon PMA activation of the neutrophil-like cells, the probe was oxidized rapidly and peaked after 20 min at an E_roGFP2_ of about -267 ± 7 mV. These kinetics were in line with observations monitoring reactive oxygen species using luminol in PMA-induced PMN [68]. The probe then showed a minor, but reproducible reduction before it reached a plateau at 30 min. In the response to reactive oxygen species, both enzymatic and non-enzymatic antioxidants are needed to maintain cellular redox homeostasis. As such, oxidants can be directly reduced by GSH, during which it is oxidized to GSSG [69]. However, oxidants, like peroxides, can be detoxified much more efficiently by enzymes, such as glutathione peroxidase, which uses GSH as the reduction equivalent [70,71]. Generated GSSG is then reduced NADPH-dependently by glutathione reductase [72,73]. Additionally, NADPH also contributes to the functional integrity of other antioxidant enzymes such as catalase and thioredoxins (Trxs) [74,75]. For instance, a CXXC motif in Thioredoxin (Trx) serves as an electron donor to reduce thiol-oxidized substrate proteins. Oxidized Trxs are subsequently reduced by TrxR in an NAPDH-dependent manner [[75], [76], [77]]. Consumed NADPH is mainly replenished in the oxidative branch of the pentose phosphate pathway (PPP), in which glucose-6-phosphate dehydrogenase (G6PDH) and 6-phosphogluconolactonase (6PGDH) are generating NADPH [78,79]. Reduction of NADP^+^ is further potentiated under oxidative stress by inhibition of GADPH that leads to the accumulation of glycolytic intermediates resulting in an increased flux into the PPP [80,81]. Taken together, it is conceivable that PMA induced generation of oxidants results initially in an increased ratio of GSSG/GSH and NADP^+^/NADPH by activation of NOX2, which also consumes NADPH, and an increase in the oxidation of protein-thiols which are then resolved by glutaredoxin and thioredoxin in an GSH and/or NADPH-dependent manner.

PMA is a pharmacological analogue of the physiological secondary messenger diacylglycerol. It was reported to activate NOX2 through a PKC-dependent pathway [82]. We assessed the response of roGFP2 after blocking NOX2 or PKC. As expected, the probe's oxidation in the cytoplasm of PLB-985 cells was abrogated. In comparison, inhibition of the PI3Ks had no effect on the probe's response, congruent with previously published reports showing that PMA-induced release of O_2_^•-^ occurred independently of PI3K [58,83]. The activation of NOX2 leads to the generation of superoxide anion, from which a mixture of different oxidants is generated, including HOCl, a highly thiol-reactive oxidant [9,49]. Inhibition of MPO, the enzyme responsible for HOCl production in neutrophils, had no effect on PMA-induced oxidation of roGFP2 in PLB-985 cells. This was somewhat unexpected, as we have shown that HOCl is the major oxidative species responsible for the probe's oxidation when expressed in phagocytized bacteria. However, PMA seems to activate NOX2 predominantly at the cell surface and not in intracellular vesicles such as the azurophil granules, in which MPO is mainly located [49,[84], [85], [86]], which could explain the lack of involvement of HOCl.

We then tested the response of roGFP2 during the phagocytosis of *E. coli*. The probe was gradually oxidized, reaching a plateau during the first 70 min at -264 ± 5 mV, in the same range as the final oxidation state achieved by PMA stimulation. A similar kinetic was observed in a previous study performed in our lab, in which roGFP2 was expressed in the cytosol of *E. coli* during phagocytosis [37]. In these phagocytized bacteria, however, the probe was oxidized to its fullest extent, unlike in the cytosol of the phagocytic cell. Quantitative thiol redox proteomics demonstrated a parallel break-down of protein thiols in those phagocytized *E. coli* cells [43]. The lesser extent of oxidation of roGFP2 in the neutrophil-like cells' cytosol suggests that these cells are able to maintain their thiol redox homeostasis. Nevertheless, stimulation by *E. coli* effectively changed the cytosolic redox potential significantly to a more oxidized state. Quantitative fluorescence microscopy revealed that roGFP2 reaches its new, higher oxidized states with substantially faster kinetics on the level of individual cells when compared to the average of the overall population. This change in the redox state presumably happens once the neutrophils phagocytized bacteria (Video 1 and Videos 2-9, Fig. 3, Fig. 4). The apparent slower overall change in fluorescence as observed in the plate reader reflects the individual cells' contribution over time to the overall more oxidized pool of roGFP2 in the sample. Phagocytosis is thus most likely the rate-limiting step for probe oxidation in PLB-985 cells, similar to roGFP2 oxidation observed in phagocytized *E. coli* [37].

Interestingly, inhibition of MPO activity by ABAH did not influence the probe's oxidation behavior in PLB-985 cells when co-incubated with bacteria. As with our observation in PMA-induced neutrophil-like cells, this is unexpected, as HOCl was the major oxidant responsible for probe oxidation in phagocytized bacteria. Especially and even more so, as HOCl-derived oxidants such as chloramines are membrane permeable [87], thiols in the cytoplasm of neutrophils should be readily oxidized, once such chlorine-containing oxidants are produced in the phagolysosome. This is in contrast to proteins and other harmful molecules that cannot permeate the membrane and, when directly released into the cytosol, would cause severe damage to the host cell [1,18,88]. Thus, neutrophils must have a highly effective defense that prevents HOCl and other reactive chlorine species from permeating the phagolysosomal membrane during phagocytosis of microbes.

We then determined the signaling pathways involved in changes of the cytoplasmic redox potential. Here, we focused on Fc*γ*-Rs mediated NOX2-activation via the tyrosine kinase Syk [89] and phospholipase C (PLC) [90] pathways. The former leads to recruitment of class I PI3Ks. Phosphoinositides produced by PI3K were shown to regulate several very distinct steps of NOX2 activation [52,91]. We found that inhibition of PI3K by the specific inhibitor Wortmannin abrogated roGFP2 oxidation in neutrophil-like cells co-incubated with opsonized bacteria. This is expected, as Wortmannin effectively inhibited ROS production in response to opsonized *E. coli* in both human and mouse neutrophils [61]. Conversely, PMA-induced oxidation of roGFP2 was abrogated by inhibition of PKC but not PI3K. PLC generates diacylglycerol (DAG) and inositol triphosphate (IP_3_) [92], which leads to the activation of protein kinase C (PKC) [54,93]. As such, PKC-δ was shown to induce oxidant production via activation of NOX2, when stimulated with IgG particles [94]. Also, when stimulated with IgG-opsonized *Aspergillus*, Gö 6983 treated human PMNs were impaired in oxidant production [95]. Similarly, opsonized *Candida albicans* induces NOX2 activation in human PMN in a PKC-dependent way [96]. Strikingly, inhibition of PKC by Gö 6983 had almost no effect on roGFP2 oxidation in neutrophil-like cells co-incubated with *E. coli* in our model, while it had a major effect in PMA treated cells. This suggests responses in neutrophils are highly tailored to the type of pathogens and the circumstances of interaction. Our results also highlight that oxidant production induced using PMA as a model for neutrophil activation, is mechanistically very distinct from activation by microbes, although the outcomes are similar. This needs to be taken into account in the interpretation of PMA-derived data.

The formation of NETs is thought to immobilize extracellular pathogens and expose them to a high dose of lethal compounds, contributing to the antimicrobial capacity of neutrophils [97]. However, a growing body of evidence suggests that different mechanisms result in NET formation depending on the stimuli [68,98]. Several studies have shown that PMA-induced NETosis depends on oxidants produced downstream of NOX2. As such, no NETs were released in CGD neutrophils upon PMA stimulation [15,99]. We showed that PLB-985 neutrophil like cells form NETs, when activated with PMA. Similar to previous studies, we could show that PMA-induced NET formation is mainly dependent on the activity of PKC, NOX2 and MPO but not PI3K [62,68,[100], [101], [102], [103]]. However, in our model, NET-formation induced by phagocytosis of bacteria was not exclusively dependent on the generation of NOX2-derived oxidants. Currently, there are a lot of discrepancies concerning the correlation of oxidant production and NET formation induced by physiological stimuli. CGD neutrophils with compromised NOX2, or neutrophils treated with DPI were shown to attenuate NET formation upon stimulation with *S. aureus* [15,104]. Nevertheless, others observed opposite effects [101,105,106]. Our results showed that DPI treatment of neutrophils inhibited the formation of bacteria-induced NET-formation only partially, despite the complete inhibition of roGFP2 oxidation. Similar to studies conducted with PMN, inhibition of MPO by ABAH significantly decreased NETosis [16,68,107] although roGFP2 oxidation in PLB-985 cells was not dependent on MPO. The most potent inhibition of phagocytosis-induced NET formation was the PI3K inhibitor Wortmannin, which is consistent with previous studies [101,108,109]. Taken together, this suggests that the observed significant change in the redox homeostasis in the cytosol of activated neutrophils is not sufficient to induce NET formation. Other (presumably also oxidative) events that do not manifest themselves in a change of the overall redox homeostasis seem to play a crucial role as well, as demonstrated by the inhibition of myeloperoxidase, which does not affect the shift in redox-homeostasis but significantly inhibits NET formation.

Our observations were made using an in vitro system, using a well-established myeloid cell line in a co-incubation assay. While we cannot say for certain if our findings can be directly applied clinically or under physiological conditions, our data suggests that oxidative signaling in immune cells has multiple layers, which lead to a shift in the cytosolic redox homeostasis, but also include highly specific signaling events required for effective microbial killing.

## Declaration of competing interest

None.
