# Supplementary material for: Activation leads to a significant shift in the intracellular redox homeostasis of neutrophil-like cells
Source: Redox Biol. 2019 Oct 13;28:101344. doi: 10.1016/j.redox.2019.101344 (PMC6807386; doi:10.1016/j.redox.2019.101344)
Supplement: Multimedia component 1 [file mmc1.pdf]

Supplementary Table 1

Primer name	Sequence
roGFP2-Orp1-BamHI-fw	CGCTGGATCCGCCACCATGGTGAGC
roGFP2-NsiI-rev	CGCTATGCATTTACTTGTACAGCTCGTCCATGC

Strain	Relevant genotype or description	Source or reference
E. coli MG1655	K-12 F^- λ^- *ilvG*$^-$ *rfb-50 rph-1*	Blattner et al., 1997 **(a)**
E. coli pCC LV	MG1655 pCC	lab collection
E. coli JM83	K-12 *ara*, Δ(*lac*-proAB), *rspL* (+strA), ϕ80, *lac*ZΔM15	Joachim Messing **(b)**
E. coli Stbl3^{TM}	HB101 F^- *mcr*B *mrrhsd*S20(r_B^-, m_B^-) *rec*A13 *sup*E44 *ara*-14 *gal*K2 *lac*Y1 *pro*A2 *rps*L20(Str^R) *xyl*-5 λ^- *leumtl*-1	ThermoFisher Scientific, Waltham, MA
E. coli AM181	MG1655 pCC_roGFP2	Degrossoli et al., 2018 **(c)**
PLB-985	Human acute myeloid leukemia cells	Drexler et al., 2003 **(i)**
HEK-293T	Human embryonal kidney cells carrying a plasmid containig SV-40 large T-antigen	DuBridge et al., 1987 **(j)**

Plasmid	Relevant genotype or description	Source or reference
pCC	TAC-MAT-Tag-2 derivative; ptac	Masuch et al., 2015 **(d)**
pASK-IBA3-mCherry	pASK-IBA3 carrying mCherry-Strep-tag®	Jan Lackmann **(e)**
pCMVR8.2	HIV-1 GAG/POL, Tat and Rev	Didier Trono **(f)**
pCMV-VSV-G	HIV-1 Env	Stewart et al., 2003 **(g)**
PLJM1-EGFP	3rd gen lentiviral vector for EGFP fusion, PGK driven puromycin	Sancak et al., 2008 **(h)**
PLJM1-roGFP2	PLM1 containing roGFP2	This work

(a) Blattner, F.R., Plunkett, G., Bloch, C.A., Perna, N.T., Burland, V., Riley, M., Collado-Vides, J., Glasner, J.D., Rode, C.K., Mayhew, G.F., Gregor, J., Davis, N.W., Kirkpatrick, H.A., Goeden, M.A., Rose, D.J., Mau, B., Shao, Y., (1997). The Complete Genome Sequence of Escherichia coli K-12. Science 277, 1453–1462.

(b) JM83 was a gift from Dr. Joachim Messing, Addgene plasmid # 50348

(c) Degrossoli, A., Müller, A., Xie, K., Schneider, J.F., Bader, V., Winklhofer, K.F., Meyer, A.J., and Leichert, L.I. (2018).
Neutrophil-generated HOCl leads to non-specific thiol oxidation in phagocytized bacteria. ELife 7 , e32288.

(d) Masuch, T., Kusnezowa, A., Nilewski, S., Bautista, J.T., Kourist, R., Leichert, L.I., (2015).
A combined bioinformatics and functional metagenomics approach to discovering lipolytic biocatalysts. Front. Microbiol. 6.

(e) pASK-IBA3-mCherry was a gift from Dr. Jan Lackmann

(f) pCMVR8.2 was a gift from Dr. Didier Trono, Addgene plasmid # 12263

(g) Stewart, S.A., Dykxhoorn, D.M., Palliser, D., Mizuno, H., Yu, E.Y., An, D.S., Sabatini, D.M., Chen, I.S.Y., Hahn, W.C., Sharp, P.A., Weinberg, R.A., Novina, C.D., (2003).
Lentivirus-delivered stable gene silencing by RNAi in primary cells. RNA N. Y. N 9, 493–501.

(h) Sancak, Y., Peterson, T.R., Shaul, Y.D., Lindquist, R.A., Thoreen, C.C., Bar-Peled, L., Sabatini, D.M., (2008).
The Rag GTPases bind raptor and mediate amino acid signaling to mTORC1. Science 320, 1496–1501.

(i) Drexler, H.G., Dirks, W.G., Matsuo, Y., MacLeod, R. a. F., 2003. False leukemia-lymphoma cell lines: an update on over 500 cell lines. Leukemia 17, 416–426.

(j) DuBridge, R.B., Tang, P., Hsia, H.C., Leong, P.M., Miller, J.H., Calos, M.P., 1987.
Analysis of mutation in human cells by using an Epstein-Barr virus shuttle system. Mol. Cell. Biol. 7, 379–387.
